# Biomechanical Evaluation of Ti-Nb-Sn Alloy Implants with a Low Young’s Modulus

**DOI:** 10.3390/ijms16035779

**Published:** 2015-03-12

**Authors:** Kenta Takahashi, Naru Shiraishi, Risa Ishiko-Uzuka, Takahisa Anada, Osamu Suzuki, Hiroshi Masumoto, Keiichi Sasaki

**Affiliations:** 1 Division of Advanced Prosthetic Dentistry, Tohoku University Graduate School of Dentistry, Tohoku University, 4-1 Seiryo-machi, Aoba-ku, Sendai 980-8577, Japan; E-Mails: kenta@dent.tohoku.ac.jp (K.T.); uzuka@dent.tohoku.ac.jp (R.I.-U.); keii@dent.tohoku.ac.jp (K.S.); 2 Division of Community Oral Health Science, Department of Community Medical Supports, Tohoku Medical Megabank Organization, Tohoku University, 2-1 Seiryo-machi, Aoba-ku, Sendai 980-8573, Japan; 3 Division of Craniofacial Function Engineering, Tohoku University Graduate School of Dentistry, Tohoku University, 4-1 Seiryo-machi, Aoba-ku, Sendai 980-8577, Japan; E-Mails: anada@m.tohoku.ac.jp (T.A.); suzuki-o@m.tohoku.ac.jp (O.S.); 4 Frontier Research Institute for Interdisciplinary Sciences, Tohoku University, Aramaki aza Aoba 6-3, Aoba-ku, Sendai 980-8578, Japan; E-Mail: hiromasu@fris.tohoku.ac.jp

**Keywords:** low Young’s modulus, Ti-Nb-Sn alloy, implant

## Abstract

Dental implants are widely used and are a predictable treatment in various edentulous cases. Occlusal overload may be causally related to implant bone loss and a loss of integration. Stress concentrations may be diminished using a mechanobiologically integrated implant with bone tissue. The purpose of this study was to investigate the biomechanical behavior, biocompatibility and bioactivity of a Ti-Nb-Sn alloy as a dental implant material. It was compared with cpTi. Cell proliferation and alkaline phosphatase (ALP) activity were quantified. To assess the degree of osseointegration, a push-in test was carried out. Cell proliferation and ALP activity in the cells grown on prepared surfaces were similar for the Ti-Nb-Sn alloy and for cpTi in all the experiments. A comparison between the Ti-Nb-Sn alloy implant and the cpTi implant revealed that no significant difference was apparent for the push-in test values. These results suggest that implants fabricated using Ti-Nb-Sn have a similar biological potential as cpTi and are capable of excellent osseointegration.

## 1. Introduction

Dental implants are widely used and a predictable treatment in various edentulous cases. However, it has been reported that various factors such as hosts, implant fixtures, surgical and prosthesis techniques influence implant failure [1,2]. Occlusal overload is causally related to implant bone loss and the loss of integration. In animals, an excessive load on implants causes marginal bone loss and crater-like bone defects [3,4].

Finite element analysis (FEA) has revealed the stress distribution of peri-implant bones. FEA requires various parameters such as implant design, the physical properties of the bone tissue, loading and boundary conditions. Nevertheless, a high von Mises stress has been observed in the cortical bone around implants because of differences in the elastic moduli and the strength of implants and tissues [5,6].

New types of titanium alloys have been developed, including Ti-29 mass%Nb (niobium)-13 mass%Ta (tantalum)-4.6 mass%Zr (zirconium) alloy and Ti-35 mass%Nb-7.9 mass%Sn (tin) alloy. These alloys consist of a β-titanium phase and are characterized by a low Young’s modulus. The Ti-Nb-Sn alloy is a novel β-type Ti alloy with a 40 GPa Young’s modulus, which is similar to that of human cortical bone (30 GPa) [7,8]. The Ti-Nb-Sn alloy is characterized by good corrosion resistance, no cytotoxicity and bone tissue compatibility [9,10,11].

We assert that the stress concentration can be diminished using a mechanobiologically integrated implant with bone tissue. To determine the effect of the low Young’s modulus, it is necessary to apply a load on Ti-Nb-Sn implants *in vivo*. However, it is not clear whether osseointegration is affected by its niobium and tin composition. Osseointegration is strongly influenced by the surface topography and the surface chemistry of dental implants. This is because the interface between the bone and the implant material is the exact site of biological reactions [12,13]. Brett *et al.* [14] reported that the surface topography of the implant material promoted the spread of the cell morphology and proliferation of the cells on the surface, and affected the expression of osteoblast genes, thus accelerating the osseointegration [14]. The purpose of this study was to investigate the biomechanical behavior, biocompatibility and bioactivity of the Ti-Nb-Sn alloy as a dental implant material.

## 2. Results

### 2.1. Surface Characteristics of the Specimens

**Figure 1:**
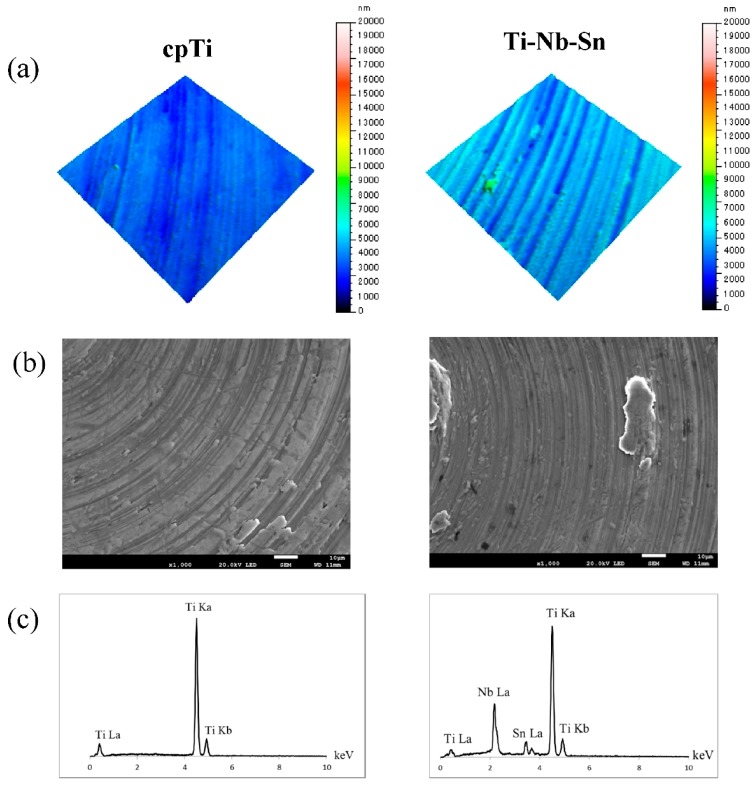
Surface characteristics of the specimens. (**a**) 3-D digital images; (**b**) SEM micrographs; and (**c**) EDX spectra.

The surface roughness was measured using a 3D non-contact surface roughness tester (Talysurf CCI6000, Taylor Hobson, Leicester, UK) and a Gaussian filter (cut-off length of 50 × 50 μm) (Figure 1a). The Sa, Sp, Sv, Sz, Sq values were 384, 4671, 2806, 7477 and 512 nm for the cpTi surface, and 577, 3975, 3789, 7764, 730 nm for Ti-Nb-Sn alloy, respectively. SEM micrographs and EDX spectra of the specimen surfaces before the experiments are shown in Figure 1b,c. The EDX spectra of cpTi and the Ti-Nb-Sn alloy only contained characteristic Ti, Nb and Sn metal peaks.

**Figure 2:**
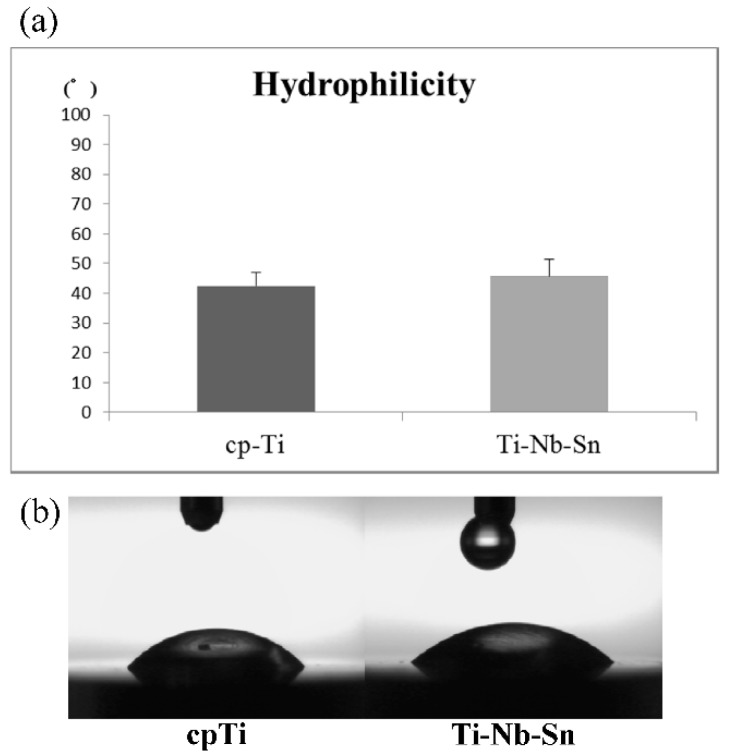
Hydrophilicity of cpTi and Ti-Nb-Sn alloy disks. (**a**) Mean contact angle of 1 μL H_2_O onto the cpTi and Ti-Nb-Sn alloy disks. Data are the mean ± SD (*n* = 8); (**b**) Photographic images of H_2_O droplets pipetted onto the cpTi and Ti-Nb-Sn alloy disks.

The hydrophilicity of cpTi and Ti-Nb-Sn alloy surfaces was measured by automatic contact angle device. The contact angles onto cpTi and Ti-Nb-Sn alloy surfaces were 42.5° ± 4.6° and 45.8° ± 5.3°, respectively (Figure 2a). Figure 2b shows the typical photography after the elapse of 10 s from dropping 1 μL H_2_O. A T-test showed no significant differences in substrate materials.

### 2.2. Cell Proliferation Assay

**Figure 3:**
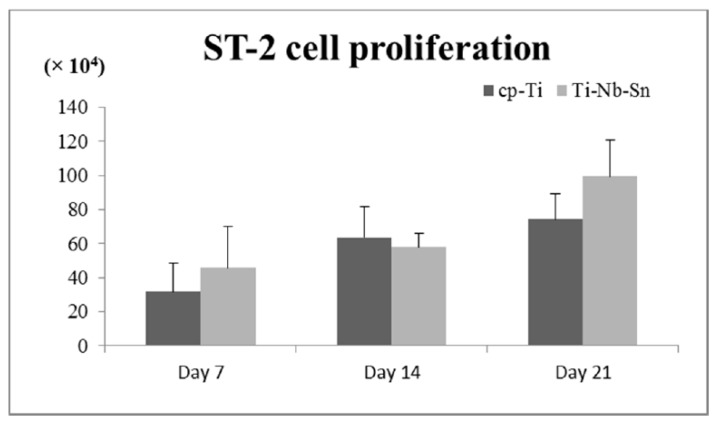
Cell proliferation assay. Data are the mean ± SD (*n* = 5).

ST-2 cell proliferation is shown in Figure 3. The number of cells (×10^4^ cells) on the cpTi and Ti-Nb-Sn alloys were: day 7; 31.8 ± 16.9, 45.7 ± 24.4, day 14; 63.7 ± 18.3, 58.1 ± 7.9, day 21; 74.4 ± 14.8, 99.3 ± 21.4. The cell proliferation assay showed that the propagated cells grew equally well on the Ti-Nb-Sn alloy and on cpTi throughout the experiments. A Tukey-Kramer test showed significant differences in the healing periods (7 day < 14, 21 and 14 day < 21 day; *p* < 0.05).

### 2.3. ALP Activity

**Figure 4:**
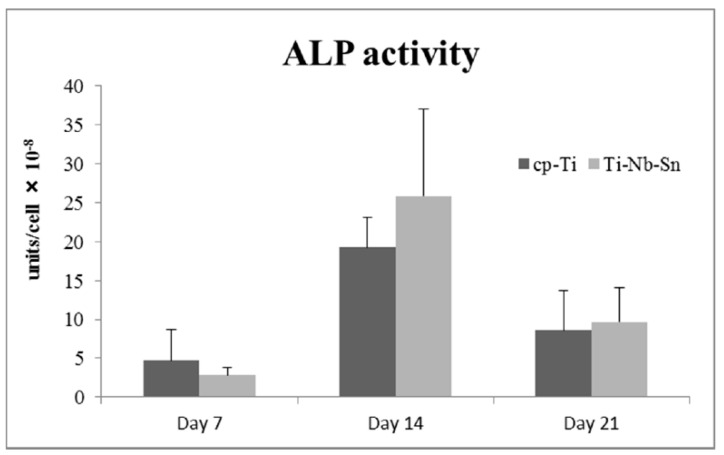
ALP activity. Data are the mean ± SD (*n* = 5).

Figure 4 shows the influence of cpTi or the Ti-Nb-Sn alloy on the ALP enzymatic activity of the ST-2 cells. The ALP activity (×10^−8^ units/cell) on the cpTi and Ti-Nb-Sn alloy were day 7; 4.7 ± 4.0, 2.8 ± 1.0, day 14; 19.3 ± 3.9, 25.8 ± 11.2, day 21; 8.6 ± 5.2, 9.7 ± 4.4. No significant difference in ALP activity was apparent in the cells grown on these surfaces. A Tukey-Kramer test showed significant differences in the healing periods (7 day < 14, 21 and 14 day < 21 day; *p* < 0.05).

### 2.4. Push-in Test Values

**Figure 5:**
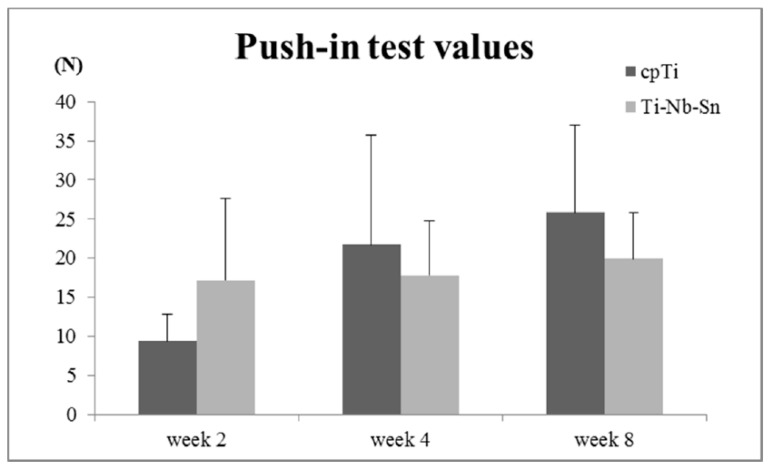
Push-in test values for cpTi and Ti-Nb-Sn alloy. Data are the mean ± SD (*n* = 5).

The obtained push-in test values are shown in Figure 5. The push-in test values (N) for cpTi and the Ti-Nb-Sn alloy were: week 2; 9.4 ± 3.4, 17.2 ± 10.4, week 4; 21.7 ± 13.9, 17.8 ± 6.9, week 8; 25.9 ± 11.1, 19.9 ± 5.9. A comparison between these groups showed no significant differences.

### 2.5. Analyses of the Implant-Tissue Interface

**Figure 6:**
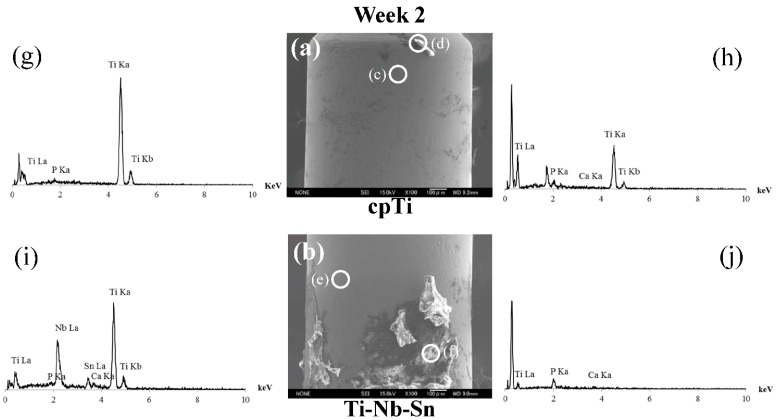
SEM micrographs and EDX spectra of excised implant after push-in tests. (**a**) cpTi; (**b**) Ti-Nb-Sn alloy are SEM micrographs at healing period week 2; (**g**–**j**) show EDX spectra, which are the EDX analyses for (**c**–**f**) in (**a**,**b**), respectively. Bars = 100 μm.

After the push-in tests, SEM observations and spot elemental analyses were performed to observe the biological structures of the excised implant surfaces. Figure 6 showed the results at week 2 of healing period. The surfaces of both substrate materials were relatively smooth and similar to those before insertion (Figure 6a,b), and metallic substrate element peaks, such as Ti, Nb and Sn, were detected (Figure 6g,i). In addition, the surfaces partially masked by remnant tissue (Figure 6d,f). EDX spot elemental analyses revealed that P peaks were present all over the surface, and Ca peaks were mainly detected at the sites with remnant tissue (Figure 6h,j).

## 3. Discussion

Many researchers have tried to use β-type Ti alloys as biomedical materials because of their regulation properties or their ability to absorb mechanical stress [15,16]. The Ti-Nb-Sn alloy was classified as a β-type Ti alloy. We assert that the stress concentration can be diminished when using a mechanobiologically integrated implant with bone tissue. The aim of this study was to confirm the biomechanical behavior, biocompatibility and bioactivity of the Ti-Nb-Sn alloy as a dental implant material and to compare it with cpTi. It was found from the results of the study that the hydrophilicity, cell proliferation, ALP activity and the push-in test values, were not significantly different between the two materials.

Osseointegration is strongly affected by the topography and chemistry of the dental implants surface because the interface between the bone and the implant material is the exact site of biological reactions [12,13]. The surface roughness (Sa) of our samples of cpTi and Ti-Nb-Sn alloy were found to be 384 and 577 nm, respectively. It has been reported that Osseointegration is likely only affected by a surface roughness of around 1 μm [17]. Guo *et al.* [18] also reported that osseointegration is affected by a 70–120 nm range difference. Our results agree with these studies. On the other hand, it was reported that nanometer ranges promoted the spread of the bone cell morphology and its proliferation, and affected the expression of osteoblast genes, thus accelerating the osseointegration [14,19]. The surface energy plays an important role in protein adsorption [20], which is evaluated by the contact angle. In this study, the contact angles of cpTi and Ti-Nb-Sn alloy were found to be 42.5° ± 4.6° and 45.8° ± 5.3°, respectively, and showed no significant differences in substrate materials. This infers that cpTi and Ti-Nb-Sn alloys might have the same protein adsorption rate, because of the protein absorption rate being correlated with the contact angle [21].

To measure the degree of osseointegration, push-in tests were employed. It has been reported that the push-in test is preferable to the pull-out test in terms of sensitivity [22]. The push-in values between two groups showed no significant differences, but the push-in values of Ti-Nb-Sn alloy were relatively higher than that of cpTi in the early stages of healing. However, it showed the opposite in the later stages. It has been reported that biomaterials, including calcium phosphate and Nb, enhance the osteogenic properties of osteoblasts [23]. Therefore, the elemental composition of Ti-Nb-Sn alloy might be affecting the bone calcification rate in the early stages.

## 4. Materials and Methods

### 4.1. Specimen Design

Commercially pure titanium Grade 2 and Ti-Nb-Sn alloy were used as substrate materials. Disks (φ8.0 mm) and cylindrical implants without screws (φ1.0 × 2.0 mm) were prepared for the *in vitro* and *in vivo* tests. These specimens were regulated at 15 μm intervals using a lathe machine. The specimens were washed with a surfactant followed by super pure water using an ultrasonic cleaner for 60 min. All the specimens underwent 15-W UV irradiation (UV cross linker FS-1500, Funakoshi, Japan) at a wavelength of 254 nm for 2 h before the experiments.

### 4.2. Surface Characterizations

The surface morphology was observed using scanning electron microscopy (SEM) and the elemental composition was examined energy-dispersive X-ray (EDX) spectroscopy (JSM-6500F, JEOL, Tokyo, Japan). The surface roughness was measured by a 3D non-contact surface roughness tester (Talysurf CCI6000, Taylor Hobson, Leicester, UK) and a Gaussian filter (cut-off length of 50 × 50 μm). The contact angle of 1 μL H_2_O droplet onto implant surfaces was measured by portable contact angle meter (PCA-1, KYOWA, Saitama, Japan) as the hydrophilicity.

### 4.3. Cell Culture and Proliferation

Mouse bone marrow stromal ST-2 (ST-2) cells were obtained from the RIKEN cell bank (Tsukuba Science City, Ibaraki, Japan). A disk substrate was used for the cell culture experiments. The ST-2 cells were cultured in α-minimal essential medium eagle (αMEM; Sigma-Aldrich, St. Louis, MO, USA) containing 1% penicillin/streptomycin (Invitrogen-Gibco, Carlsbad, CA, USA) and 10% Fetal Bovine Serum (FBS; life technologies, Carlsbad, CA, USA) under 5% CO_2_ and at 37 °C. The disks were placed in ultra-low attachment surface 24-well plates (Sigma-Aldrich, St. Louis, MO, USA). 10 × 10^4^ ST-2 cells were seeded onto each disk and the plates were incubated for 7, 14 and 21 day. The proliferation of cells on the disks was quantified by WST-8 colorimetry (Dojindo Laboratories, Kumamoto, Japan).

### 4.4. Alkaline Phosphatase (ALP) Activity

ALP activity was measured by a colorimetry-based assay (LabAssay ALP; Wako Pure Chemical, Osaka, Japan) using *p*-nitrophenylphosphate as the substrate. The ALP activity was normalized using the cell numbers from a WST-8 assay.

### 4.5. Animal Experiments

All procedures were approved by the Animal Research Committee of Tohoku University.

Ten-week-old male Sprague-Dawley (SD) rats weighting 280–300 g were prepared. The experimental rats were inducted by isoflurane anesthesia (2.0%, 4 L/min), which was maintained (1.5%, 4 L/min). A full-thickness incision was made to give a 50 mm straight line from the inside of a knee joint to the hip joint neighborhood. The animals were exfoliated at the skin and the periosteum and the exposed thighbone were at the surface. With irrigation, an implant cavity was prepared using a pilot drill 7 and 11 mm from the distal edge of the femur. The joint capsule and the muscle were sewed with a suture needle of 4-0 silk after washing with a saline solution. The skin sutures were sewed using a 5-0 polyglycolic acid suture thread. These rats were sacrificed after healing periods of 2, 4 and 8 weeks and the bones containing the implants were harvested.

### 4.6. Push-in Tests

Push-in tests illustrate the degree of osseointegration as reported by Ogawa, *et al.* [24]. The femurs within the implants that were harvested were immediately embedded in autopolymerizing resin (FIXPEED, GC, Tokyo, Japan). Push-in tests were carried out using a universal testing machine (EZ-L-500N, Shimadzu Corporation, Kyoto, Japan). The resin block was positioned using a 3D adjustable table to load the implant vertically. The push-in test value is defined as the breakpoint load at the implant-tissue interface.

### 4.7. Analyses of the Implant-Tissue Interface

After the push-in tests the excised implants were soaked in stirred distilled water for 1 h and dried at 37 °C under vacuum. The implant surfaces after the push-in tests were compared using SEM and EDX spectroscopy.

### 4.8. Statistical Analysis

Number of samples was 8 for H_2_O contact angle, and examined the differences in substrate materials by the independent samples *t*-test; *p* < 0.05 was considered significant. Five samples (*n* = 5) were used for the cell culture studies and the push-in tests. A two-way ANOVA was used to examine the effect of the healing periods and the difference in substrate materials. When necessary, the *post-hoc* Tukey-Kramer test was used; *p* < 0.05 was considered significant.

## 5. Conclusions

This is the first study that evaluates implants fabricated from Ti-Nb-Sn with a low Young’s modulus from the aspect of the biomechanical behavior, biocompatibility and bioactivity. Our results suggest that Ti-Nb-Sn implants have a similar biological potential as cpTi, and they have excellent osseointegration.
